# Impacts of Built Environment on Urban Vitality: Regression Analyses of Beijing and Chengdu, China

**DOI:** 10.3390/ijerph16234592

**Published:** 2019-11-20

**Authors:** Shiwei Lu, Chaoyang Shi, Xiping Yang

**Affiliations:** 1 Key Laboratory of Urban Land Resources Monitoring and Simulation, Ministry of Land and Resources of China, Shenzhen 518034, China; lusw_gis@163.com; 2 School of Architecture and Urban Planning, Huazhong University of Science and Technology, Wuhan 430074, China; 3 School of Civil Engineering and Mechanics, Huazhong University of Science and Technology, Wuhan 430074, China; 4 School of Geography and Tourism, Shaanxi Normal University, Xi’an 710119, China; xpyang@snnu.edu.cn; 5 Geomatics Technology and Application key Laboratory of Qinghai Province, Xining 810001, China

**Keywords:** social media check-in data, built environment, urban vitality, heterogeneous patterns, regression analyses

## Abstract

The loss of urban vitality is an important problem in the development of urban central areas. Analyzing the correlation between urban built environment and urban vitality supports urban planning and design. However, current research excludes the study of how consistent built environment factors affect urban vitality of cities with different development situations. Therefore, using social media check-in data, this paper measures neighborhood vibrancy in urban central areas in Beijing and Chengdu, China. Four levels of spatial information were used to measure the built environment: regulatory planning management unit (RPMU), land use, road network, and building. Regression model is used to quantify the correlation between urban vitality and the built environment of these two cities. The study found a strong correlation between built environment factors and urban vitality. Among the built environment factors, points of interest (POI) diversity and public transport accessibility indicators were strongly positively correlated with neighborhood vibrancy. However, the density indicators had totally different effects on urban vitality of cities with different development situations, which is excluded in existing studies. This research strengthens the practical understanding of the compact city concept, and can support the design and planning of urban built environment.

## 1. Introduction

Creating continuous urban vitality is critically important for developing urban central areas [1], which could contribute to high concentrations of people, information, and capital flow to urban central areas. When the use of high-quality urban infrastructure and resources is not maximized, the urban space can become monotonous and undifferentiated, leading to a ghost city phenomenon [2]. Urban central areas have experienced the significant problem of having a lack of vitality, which can increase the risk of night crimes [3]. Therefore, it is important to understand the current dynamic of urban vitality and to explore the creation and internal mechanisms of urban vitality. This understanding can help improve the quality of urban space during the practice of urban planning and construction. This paper analyzes the relationship between built environment and the neighborhood vibrancy of cities. The goal is to evaluate the impact of built environment factors on neighborhood vibrancy, to enhance urban vitality.

Since the 1950s, Western scholars have consistently applied modernist urban theory in the practice of urban planning and design, and the study of urban space has become more thorough [4,5]. People-oriented, diverse, and vibrant urban space has received increased attention; examples include Jane Jacobs’s theory of diversity [4], Norberg-Schulz’s space and place theory [6], and Jan Gael’s theory of public communication [5]. These have established a rich theoretical foundation for studying contemporary urban vitality. Bentley et al. proposed that survivability, safety, and adaptability are necessary conditions for cities to survive and develop [7]. They emphasized the importance of functional diversity in urban life. Both Jan Gehl and Jane Jacobs noted that people and space for their activities make a city vibrant [4,5].

However, it was not until the early 2000′s that Jane Jacobs’s concept of urban vitality and diversity for urban planning and design received much attention in China [4]. Urban forms characterized by high-density and compact intensity have been more respected, as they are considered effective in curbing urban sprawl, and have multiple advantages such as saving natural resources and improving the efficiency and quality of space utilization [8]. Jacobs noted that urban vitality needs to be explored at the urban streets and neighborhood level [4]. The spatial characteristics and social attributes of the inner blocks of Chinese cities differ significantly from those of western countries. China’s urbanization is currently characterized by rapid and low-quality urban expansion, while the concept of small block and dense road network was highly recommended in many western countries [2]. The comprehensive and refined measurement of spatial patterns of Chinese cities has attracted significant attention [9,10].

Studies have highlighted that built environment indicators, including land use, accessibility, and building density have profoundly affected the creation of urban vitality [11,12]. However, some scholars have begun to question the effects of construction density and accessibility on the creation of neighborhood vibrancy [13,14]. The reason for the inconsistency in different conclusions may be the imperfection of built environment measurement system, or the indeterminacy of development stage and construction situation of different cities. How the consistent built environment factors affect urban vitality of cities with different development situations is excluded in current researches. Therefore, it is important to conduct comparative analysis of built environment measurements for different cities with different development stages to evaluate urban vitality. This comparative approach could be used to guide the construction of emerging cities.

Traditional research has mainly used surveys or questionnaires. In this traditional data environment, built environment measurements and vibrancy evaluation lack the support of data and quantitative methods. Today’s information and communication technology (ICT) and Internet technologies have initiated tremendous changes in people’s lifestyles and methods for organizing cities. First, the availability of big data makes it possible to collect highly precise long-term sequences of human mobility data, including social network data, global positioning system (GPS) data, bus credit card data, and mobile phone data [15,16,17]. Big data can describe the distribution of human activities during normal and big events. Second, many map platforms provide free urban spatial information data, including points of interest (POIs), road networks, and building vectors. These provide the data foundations to quantitatively measure built environment.

This study applied residential activity information and urban spatial basic information provided by different open data platforms to quantitatively explore the impact of urban built environment on urban vitality. This study makes two primary contributions. First, using open data platforms, the study quantitatively measured urban vitality and the built environment of two cities with different development situations. Second, the study included a quantitative and comparative analysis of the correlations between built environment and neighborhood vibrancy of these two cities. Last and most important, the study found that the same built environment factors could contribute to contrary effects on urban vitality of cities with different development situations, which is excluded in existing studies. Through comparative analysis, the study’s conclusions may provide better guidance for urban planning practice and improving urban vitality for large amounts of emerging cities in China, such as avoiding disorderly city expansion and over-exploitation simultaneously.

The rest of this paper is structured as follows. Section 2 provides a literature review about urban vitality and built environment. Section 3 introduces the research area and data used for this study. Section 4 and Section 5 introduce the measurement system and methodology. Section 6 focuses on the results of the spatial regression analysis. Section 7 makes a discussion about this research, and Section 8 summarizes and concludes the research.

## 2. Related Work

### 2.1. Urban Vitality

Creating urban vitality has long been a focus for urban planners. Jan Gehl and Jane Jacobs both proposed that urban vitality stems from the people and their activities in a space [4,5]. Urban vitality affects resident health [18], urban public safety [18,19], socioeconomic development and spatial linkages [20], and urban space quality [21]. Kevin Lynch proposed measuring urban spatial form and value by using five indicators: vitality, feeling, suitability, accessibility, and management. The definition of vitality is the level of support for life and requirements for ecology and human beings [21]. Gehl [5] and Attoe & Logan [22] argued that behaviors associated with daily life in cities are the foundation and starting point of urban vitality. These important theories have laid a rich theoretical foundation to study urban vitality.

Evaluating urban vitality in the traditional data environment is mostly based on qualitative perspectives, or through field observations and questionnaires. For example, March et al. [23] pointed out that measuring vitality should consider the different experiences required for a healthy life, including privacy, rest, and contemplation. Sung & Lee [24] conducted a telephone survey to study the daily walking activities of Seoul residents, further revealing the connection between the residential environments and walking activity. However, acquiring these data is time-consuming and laborious, and the limited representativeness of data limits the depth and breadth of urban vitality research.

Urban vitality can also be characterized from the perspectives of employment data, economic development level, and cultural exchange [25,26,27]. From a residential movement perspective, urban space vitality reflects the diversity of urban life produced by human convergence and activities [28]. Fortunately, the rapid development of information & communication technology (ICT) has enabled the acquisition of daily activity data for a large number of residents. Many scholars currently mainly obtain information about the spatial distribution of residents through mobile phone signaling data, Location-based service (LBS) data, and GPS tracking data. They then extract the population distribution to study the temporal and spatial characteristics of urban vitality [15,16,29]. Yue et al. [15] noted that neighborhood vibrancy can be assessed by the number of active people in the neighborhood, extracted from mobile phone location data.

In addition, urban dynamics differ in different time periods. In the nineteenth century, human society facilitated the use of light at night to maximize working hours and intensity [30]. Spatial-temporal big data can be effectively used to study human mobility patterns under different time units. Ratti [31] used mobile phone signaling data to study the temporal rhythm of urban activities in Milan, Italy. This was done by comparing the spatial distribution of mobile phone users and the intensity of their activities in different time periods. Lee et al. [32] used mobile phone location data to analyze hourly resident activity patterns and urban spatio-temporal expansion characteristics. They extracted temporal and spatial variation of activity centers and hotspot regions. In addition, Wu et al. [16] also noted inconsistencies in the spatial diversity characteristics of urban vitality between Shenzhen’s working days and weekends. However, the study of urban vitality has not been addressed in the current research and should be explored.

In summary, the urban dynamics and vitality in different periods display different characteristics, and quantitatively evaluating urban vitality is not an easy task. From the perspective of the real-time activities of residents, the existing research has not addressed the current situation of urban vitality as a result of big events, and has not explored the creation factors and internal mechanisms of urban vitality. This article applies a new data source to study the urban vitality of all the neighborhoods in the central urban areas of Beijing and Chengdu, China.

### 2.2. Built Environment and Urban Vitality

Urban form profoundly affects the health of a city, the level of economic development, and city sustainability [33]. Studies about urban forms have been mainly done by scholars in urban geography, urban planning, architecture, and landscape design. An early definition of urban form was provided by Schlüter [34], who defined the term as a trace of human behavior left on the earth’s surface, consisting of elements such as land, settlements, traffic lines, and surface buildings. Conzen further developed the concept of urban morphological genes [35]. Bourne [36] defined urban form as space, topography, and internal form (including density, heterogeneity, organizational principles, and social behavior). New Urbanism advocates the reintegration of spatial form and constructed environments to form a perfect city and neighborhood unit [37]. The compact city concept advocates the need to save and intensively use land resources in urban planning, centralize urban functional elements, and strengthen urban space growth management [8].

Based on these concepts and theories, quantitatively measuring the spatial patterns of urban forms is important for studying the associations between urban forms and other urban problems. Song et al. [11] proposed 27 sets of urban form measurements from three perspectives: permeability measures, vitality and accessibility measures, and variety measures. Ye & Van [38] used geographical information system (GIS) to integrate different frameworks and indicators of urban form and to measure urban spatial quality. Yang et al. [39] explored human mobility hotspots and patterns in different land use properties. Yang [40] found that socio-economic data and land use affect the travel patterns of older people in Hong Kong. These studies lead to the conclusion that urban vitality can be created using design methods. Exploring the relationship between built environment and urban vitality helps create better urban space.

Katz [41] noted that the degree of compactness, walking scale, functional mixing, and appropriate building density are important factors influencing urban vitality. Jacobs [4] posited that functional mixing, pedestrian blocks, mixing of old and new buildings, and dense population distribution are necessary to maintain urban vitality. Montgomery [1] proposed that vibrant urban space should have a detailed texture, humanity scale, mixed function, and street accessibility. Attoe & Logan [22] proposed urban catalyst theory, noting that buildings, places, and areas can become popular and drive elements of neighboring cities. Adedeji [42] listed factors that characterize the quality of public landscapes, including visual accessibility and satisfaction, aesthetics, cleanliness and visual quality, and open space for easy access.

Studies generally divide urban space accessibility into visual accessibility, physical accessibility, and symbolic accessibility [43,44,45]. Gehl [5] pointed out that richness of street interfaces, the diversity of urban public facilities, and the diversity of activity space are important conditions for urban public life. Ewing et al. [46] listed nine qualities that influence urban walking behavior: imagery, fitness, scale of humanity, transparency, richness, ease of identification, consistency, continuity, and cleanliness. Clark et al. [47] used urban shape, road density, population density, and population concentration as the main indicators of built environment to study the relationship between air quality and built environment in the United States. Yue et al. [15] explored the relationship between neighborhood vibrancy and the mix and diversity of POIs using linear regression models; however, the study did not extend to analyzing the interaction mechanism between different built environment factors.

A summary of the theoretical results on the connotation and creation principles of urban vitality indicates there is no consensus on the impacts of built environment. Further, the selected indicators differ. Based on research by different scholars, the built environment that affect space vitality can be classified into six perspectives: the space function and use, accessibility, intensity and density, shape and scale of space, the landscape, and the location in the spatial and social environment [1,4,5,41,48]. There is a lack of urban spatial information data. Most studies have qualitatively explained the correlation between urban vitality and built environment factors. In contrast, their quantitative correlation needs further discussion. Quantitative measures and analytical methods, such as multiple regression analysis, are trends that reveal the associations [29,49]. In addition, researchers have not yet assessed the correlation between urban vitality and built environment of cities with different scales and development situations.

Therefore, this study used urban spatial information data obtained from different platforms to measure the built environment of neighborhoods in the central areas of Beijing and Chengdu, China. The goal was to explore the quantitative relationship between the consistent built environment and urban vitality of cities with different scales and development situations, to answer how the consistent built environment factors affect urban vitality of different cities.

## 3. Study Area and Data Sources

### 3.1. Study Area

The cities studied in this study included Beijing and Chengdu, China. This study first describes the research areas of these two cities.

Beijing is the capital city of China. Its national central city index ranks first in China. In 2017, the resident population of Beijing was 21.7 million, and the gross domestic product (GDP) exceeded 2.8 trillion yuan, ranking second in China’s urban GDP. The central area of Beijing includes six districts: Dongcheng District, Xicheng District, Chaoyang District, Fengtai District, Shijingshan District, and Haidian District. These districts account for 8 percent of the total area of Beijing (about 1312.8 square kilometers). The spatial analysis units for this paper included 113 regulatory planning management unit (RPMU) in these six districts.

Chengdu is the capital city of Sichuan Province, and has more than 16 million total residents. In 2017, its GDP exceeded 1.3 trillion yuan, ranking eighth in China’s urban GDP. Chengdu is an important central city in western China, with an urbanization rate of 70.6%. The central urban area of Chengdu includes Chenghua District, Wuhou District, Jinniu District, Jinjiang District, and Qingyang District. These five districts are the most prosperous and oldest districts in Chengdu. The five districts cover an area of approximately 420 square kilometers. The study evaluated 76 regulatory planning management unit (RPMU) in the five major districts.

**Figure 1:**
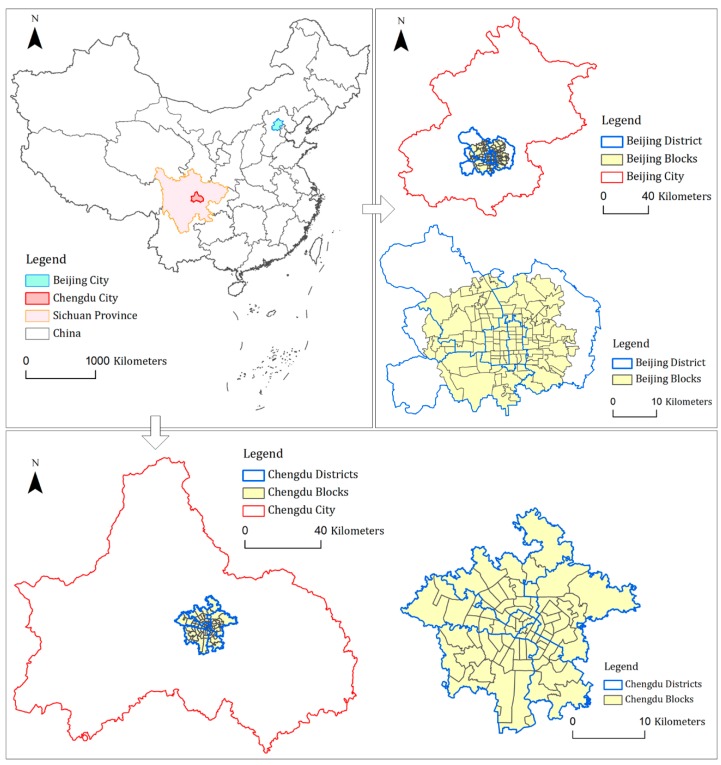
Study areas of this research.

Beijing is one of the four biggest cities in China (other three biggest cities are Shanghai, Guangzhou, and Shenzhen), and has become an international metropolis and well-urbanized. The characteristics of urban built environment in the central urban area of Beijing will not change much. However, Chengdu is a typical representative of the emerging cities in China, and facing tremendous constructions and changes. Chengdu is expected to become another international metropolis. The development and construction situations of these two cities are totally different. How the same built environment factors affect urban vitality of cities with different development situations needs deeply studied. Thus, the comparative analysis of these two cities is helpful to better guide the construction and planning of these emerging cities in China to avoid disorderly city expansion and over-exploitation. The basic spatial analysis unit in this study is the urban RPMU in the central areas of Beijing and Chengdu. Figure 1 shows the spatial locations.

### 3.2. Data Sources

The data used for this study included social-media check-in data as resident activity data, socio-economic data, and urban spatial information data. The urban spatial data included information on the city’s regulatory planning management units (RPMUs), POIs for land use function, road network (including public transportation sites), and building information vector data.

#### 3.2.1. Social-Media Check-in Data

*Sina* micro-blog check-in data were used to represent neighborhood vibrancy. The *Sina* micro-blog is similar to Twitter and Facebook, and is one of the largest social media platforms in China. The platform has 165 million daily active users, and the check-in location is accurate to the meter level. Data collection was facilitated by the *Sina* micro-blog application program interfaces (APIs). Through the “place/users/checkins” API (https://open.weibo.com/wiki/2/place/users/checkins), we retrieved the list of places where users have checked in. The check-in data obtained for this study covers the period from September 1 to 7, 2016. A total of 124,658 locations were recorded in the 113 RMPU in the central areas of Beijing, and a total of 50,719 locations were recorded in the 76 RMPU in the central urban area of Chengdu.

#### 3.2.2. Socio-Economic Data

The socio-economic data used for this study mainly included resident population data and house price data. The resident population refers to the number of people who actually live in the city for more than half a year. This indicator is different from the Hukou population, who may not live in that city. The resident population better reflects the actual population distribution of the city.

Resident population data were obtained by reviewing the yearbooks in each city (http://www.cdstats.chengdu.gov.cn/ and http://www.bjstats.gov.cn/index.html). Housing price data were obtained from Lianjia, one of China’s largest rental and sales platforms (https://www.lianjia.com). Previous studies have shown that the spatial convergence of people is closely related to housing prices [50].

#### 3.2.3. Point of Interest (POI)

**Figure 2:**
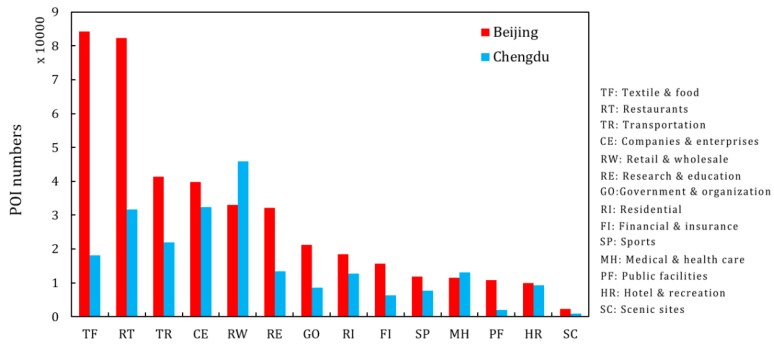
Number of points of interest (POIs) in each category of Beijing and Chengdu.

The POI data used in this study were collected from the Amap, one of China’s largest map search engines and suppliers. Amap is affiliated to AutoNavi company, which is located in Beijing. Amap provides free application interfaces to enable data collection from different layers and features. The labels for the POI data addressed all types of facilities. A total of 223,595 POIs were obtained in the central areas of Chengdu, and 414,425 POIs were obtained in central area of Beijing. These POIs belong to 14 categories: textile & food, restaurants, transportation, companies & enterprises, retail & wholesale, research & education, government & organization, residential, financial & insurance, sports, medical & health care, public facilities, hotel & recreation, and scenic sites. Figure 2 shows the distribution of POIs for each category in Beijing and Chengdu.

There were significantly more POIs at each category in Beijing than in Chengdu, especially in the textile & food and restaurants categories. There were fewer retail & wholesale POIs in the central urban area of Beijing than in Chengdu. This may be because there are government departments, a large number of courtyard houses and old buildings with a long history in the central urban area of Beijing. Retail & wholesale locations are much more strictly regulated.

#### 3.2.4. Building Vector Data and Road Network Data

This study used building vector data (including building height) to calculate the construction intensity and building density in each neighborhood. Road network data were downloaded from Openstreetmap (https://www.openstreetmap.org/), which provides detailed road vector information for global users.

## 4. Definitions of Variables

This study applied social-media check-in data to measure neighborhood vibrancy, introduced socio-economic indicators, and measured the built environment from five major systems: spatial accessibility, spatial intensity and density, spatial mixing function, spatial shape compactness, and landscape [1,4,5,41,48].

### 4.1. Neighborhood Vibrancy Measurement (Dependent Variables)

Yue et al. used the total accumulated population to quantify the neighborhood vibrancy in Shenzhen, thus, this study used the accumulated number of people who checked-in at each neighborhood to measure vibrancy *v_i_* [15]. Compared to traditional questionnaire data, social media check-in data provides a higher sampling rate and can be used to characterize urban vitality [16]. The higher the number of check-in records in the RMPU, the more people are likely to be active in the RMPU, that is, the higher the vibrancy of RMPU.

### 4.2. Social-Economic Indicators

Jacobs [4] noted that good urban vitality requires a dense population. The population indicators in this study mainly included the resident population in each RMPU. The study used the average housing price in each RMPU as the economic indicator.

### 4.3. Accessibility Indicators

Public transport accessibility mainly refers to the distribution of bus and metro stations within the RMPU. This paper uses the density of bus stations (*BSI*) in the RMPU to measure the degree of bus accessibility.

The road density index (*RDI*) measures the degree of road density in a neighborhood. The *RDI* further describes the construction strength and accessibility of transportation facilities and services. For this study, *RDI* was calculated as:

(1) $$RDI_{i}=L_{i}/S_{i}$$

Here, *S_i_* is the area of neighborhood *i*; *L*_i_ is the total length of the center lines of the roads in neighborhood *i*.

### 4.4. Density and Construction Strength

Many indicators are used to measure a neighborhood’s construction strength and density. However, construction strength and density are mainly based on the neighborhood’s own characteristics, construction intensity, and degree of mixed function [14]. Studies have shown that good building environment requires suitable spatial construction strength and density.

First, this research introduces the floor area ratio (*FAR*) to measure the level of spatial construction strength. The *FAR* is an important economic and technical indicator reflecting the intensity of urban construction. The larger the *FAR* is, the greater the construction intensity is, and the higher the degree of land use is. This study calculated the *FAR* as:

(2) $$FAR_{i}=Q_{i}/S_{i}$$

Here, *Q_i_* is the total construction area of neighborhood *i*, including the ground and aerial construction area.

Second, this research introduces the building density index (*BDI*) to measure the level of ground construction strength. In this study, *BDI* is the ratio of the projected area of buildings to the area of the neighborhood. This indicates the land occupation rate of that neighborhood, as well as the intensity of building coverage. BDI was calculated as:

(3) $$BDI_{i}={M_{i}/S}_{i}$$

Here, *M_i_* is the total base area of all buildings in neighborhood *i*.

### 4.5. Mixed Function

The spatial function focuses on land use type and its degree of mixing, and is a key indicator when measuring urban spatial diversity. A number of studies have indicated that the diversity of POIs represents the degree of the mixed function in an urban space [15]. Based on the information entropy calculation method, the Shannon-Weaver diversity index was used to calculate the POI diversity index in each neighborhood. The index was calculated as follows:

(4) $$Entropy=-\sum_{j=1}^{n}p_{j}log_{2}p_{j}$$

Here, *n* is the number of POI types, and the *i*th POI has a relative proportion of *p_j_*.

### 4.6. Shape Indicators

For this study, Area (*S_i_*) and the Richardson compactness index (*RCI*) are used to measure the shape indicators of each neighborhood. The compactness index uses the circular region as the standard unit measure of shape compactness with a value of 1. The compactness of other regions is less than 1. That is, the smaller the compactness index value, the greater the dispersion of the urban form and the less compact the urban space. The Richardson compactness index (*RCI*) was calculated as:

(5) $$RCI_{i}=\frac{\sqrt{\pi S_{i}}}{C_{i}}$$

Here, *C_i_* is the perimeter of neighborhood *i*.

### 4.7. Landscape Quality Indicator

In terms of green landscape, this study used the Green Coverage Index (GCI) to measure the quality of green landscape in each RMPU. GCI is measured by the ratio of large-scale ecological green land and park area (*G_i_*) in the RMPU.

(6) $$GCI_{i}=\frac{G_{i}}{S_{i}}$$

## 5. Methodology

### 5.1. Spatial Autocorrelation

In this paper, the Global Moran’s I is used to calculate the potential interdependence of spatial vitality between different RMPU. The Global Moran’s I is calculated as:

(7) $$I=\frac{n}{\sum_{i=1}^{n}\sum_{j=1}^{n}w_{i,j}}\cdot \frac{\sum_{i=1}^{n}\sum_{j=1}^{n}w_{i,j}z_{i}z_{j}}{\sum_{i=1}^{n}z_{i}^{2}}$$

Here, *z_i_* is the deviation between the vibrancy of *i*th RMPU and the mean value of vibrancy of all RMPU, *w_i,j_* is the spatial weight between *i*th and *j*th RMPU, which is measured by the reciprocal distance between the centers of these two RMPU. The above spatial autocorrelation analysis can be completed in the spatial statistics toolbox of ArcGIS version 10.2.2. ArcGIS is a software produce of ESRI company, which is located in California, USA.

### 5.2. Regression Model

The linear regression model was used to explore the comparative analysis between urban built environment factors and urban vitality of Beijing and Chengdu [15]. The dependent variable is neighborhood vibrancy, as measured by check-in data in Section 4.1. Independent variables include socio-economic factors and built environment indicators. Socio-economic factors (populations and economic data) are control variables. In linear regression model 1, these socio-economic variables are introduced to explain any variation in neighborhood vibrancy that is not related to the built environment [15]. Linear regression model 2 included built environment factors as additional independent variables to future explore the impacts of built environment on urban vitality.

## 6. Results and Analysis

### 6.1. Variable Statistics

Table 1 shows the statistics for the socio-economic indicators and built environment indicators for the two studied cities.

As the capital city of China, Beijing has a larger RMPU area and population density than Chengdu. The biggest difference across the indicators was housing price and bus station density. Beijing’s housing prices are approximately five times larger than Chengdu’s. In terms of accessibility indicators, Beijing’s bus station density indicator is approximately three times larger than that of Chengdu. Differences in the spatial distribution of the indicators could contribute to the variations in neighborhood vibrancy.

### 6.2. Spatial Autocorrelation Analysis of Neighborhood Vibrancy

**Figure 3:**
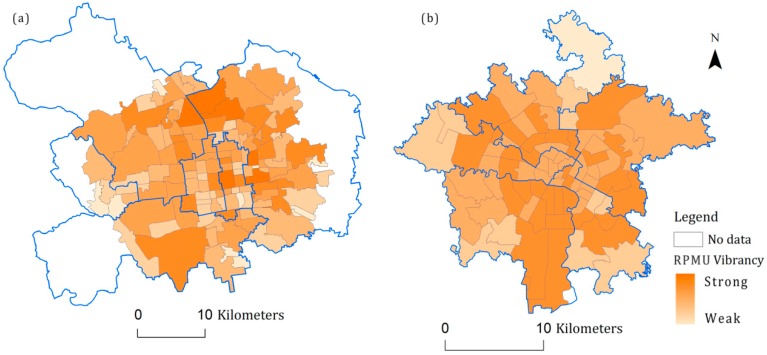
Spatial distribution patterns of neighborhood vibrancy. (**a**) Beijing, (**b**) Chengdu.

Figure 3 shows the neighborhood vibrancy for Beijing and Chengdu. The vibrancy scale in these two maps are the same. The figure shows that the neighborhood vibrancy of Beijing’s central urban district was significantly stronger than Chengdu. The number of people who checked in at the Beijing neighborhoods reached 10,000; the number of people who checked in at the Chengdu neighborhoods was less than 2000. This may be because that the total population for the different cities, the check-in preference of residents, and number of people attracted from other cities could all impact the quantitative results of the neighborhood vibrancy. In addition, neighborhood vibrancy in the central urban areas of the two cities showed clear spatial heterogeneous patterns, indicating differences in the spatial vibrancy of each RMPU. The common feature of the two cities is that the vibrancy levels in the most central areas were not the highest of all the studied area neighborhoods. The eastern and northern parts of Beijing were more vibrant, followed by the south and the west. The most vibrant area of Chengdu was the ring-shaped area radiating outward from the most central area.

A spatial autocorrelation analysis was conducted to assess the neighborhood vibrancy of Beijing and Chengdu. The global spatial autocorrelation coefficient of Chengdu was 0.04; the value for Beijing was 0.07 (*p*-value = 0.01). The correlation result was very close to 0, further indicating that neighborhood vibrancy was neither clustered nor dispersed. The heterogeneous patterns of neighborhood vibrancy in the study areas were very strong. In summary, the spatial heterogeneous of neighborhood vibrancy and built environment indicators suggested that different built environment may contribute to different vibrancy. This provided the application basis for spatial regression analysis.

### 6.3. Spatial Regression Results and Analysis

To describe the relationship between the built environment indicators of different cities and neighborhood vibrancy, two sets of linear regression analysis were conducted for the two cities. In the first set of regression models, only socio-economic indicators were considered to explain the variances of neighborhood vibrancy. These indicators were directly unrelated to the built environment. Table 2 shows the detailed results of the linear regression between selected indicators and neighborhood vibrancy. The variance inflation factor was less than 3 for all variables. This indicates that the variables are not redundant; therefore, multicollinearity should not impact analysis results.

In model 1, both the regression results for the two cities showed that socio-economic indicators account for approximately 30% of the neighborhood vibrancy. In particular, there was a negative correlation between population density and neighborhood vibrancy. This result differed from Jacobs’s suggestion that good neighborhood vitality requires a dense population distribution [4]. This study result may have occurred because the resident population density in the inner RMPU of the central areas of Beijing and Chengdu was excessively large, exceeding 20,000 people per square kilometer. Residents may be more inclined to conduct social media check-in activities in moderately dense or culturally rich places. In addition, there was a strong positive correlation between housing prices and neighborhood vibrancy, particularly for Chengdu. Studies have found the spatial convergence of people is closely related to housing prices [50]. Housing prices can also indirectly reflect local economic and consumption levels, and are directly related to spatial location. Prices are higher for homes close to convenient transportation, parks, and large commercial districts. These geographically superior locations are also destinations to which residents prefer to travel. Thus, there is a high possibility of check-in behavior, and the neighborhood is considered more active.

In model 2, the built environment factor is introduced into the regression analysis, significantly improving model accuracy. This indicates that there is a strong correlation between built environment indicators and neighborhood vibrancy. For Chengdu, the *R^2^* increased from 0.27 to 0.55; for Beijing, *R^2^* increased from 0.31 to 0.50. In addition, the model intercept dropped from 3.96 to 2.12 and from 4.74 to 3.08 for Beijing and Chengdu, respectively. This indicates a gradual improvement in regression model performance. The *R^2^* value is only about 0.55 in these models; however, the p-value is significant at 0.05. This indicates that the two linear regression models are statistically significant in illustrating patterns of neighborhood vibrancy.

Commonly, indicators such as house prices, entropy of function diversity, and transport accessibility are highly positively correlated with neighborhood vibrancy. Other studies have also validated the role of function diversity in creating good vibrancy [15]. Jacobs [4] noted that diversity is the nature of city, meeting the diverse needs of different groups of people. The compact city also advocates a multi-purpose, mixed-function area to replace the traditional, relatively single-use functional area. This could stimulate the vitality of urban space.

The bus stations density indicator in the RMPU plays a significant role in promoting neighborhood vibrancy, especially for Chengdu. Chengdu has a bus station density that is only one-third of Beijing. The higher the spatial accessibility, the higher the connectivity of places with other urban places. Residents can more easily move from the surrounding area for activities and social interactions, inspiring neighborhood vibrancy.

In addition, the compactness of RMPU shape has a significant impact on a RMPU’s vitality. Table 1 showed that the compactness distribution of RMPU shapes of Beijing and Chengdu were roughly similar; both were less than 0.5. The RMPU areas in this study were large, with average areas of RMPUs in Beijing and Chengdu being 6.69 and 5.97 km^2^ respectively. There was an indirect jaggedness of the RMPU boundary, impacting the orientation of internal buildings and the constraints of the road inside the RMPU. This affects check-in behavior.

The *FAR* and building density indicator are two common indicators for characterizing the strength of spatial and ground construction. However, they appear to have played different roles in the creation of neighborhood vibrancy in Beijing and Chengdu. For Beijing, the *FAR* was positively correlated with neighborhood vibrancy, whereas building density indicator was negatively correlated. For Chengdu, *FAR* was negatively correlated with the neighborhood vibrancy, whereas building density had a strong positive correlation. Beijing is one of the most developed cities of China, and the city has a large number of low-floor historical architectures and modern buildings. The average *FAR* of neighborhoods in the central areas of Beijing was less than 1, indicating the construction pattern was characterized by high ground construction intensity, but the building floor was very low. However, Chengdu was different. The average *FAR* of the central area was approximately 1.80. The building floor in central area was higher than Beijing. Thus, it is important to coordinate the relationship between building density and *FAR*, that is, to control the ground construction intensity and floor height. Urban development should avoid disorderly expansion and over exploitation. Finally, the green coverage index also plays a positive role in creating neighborhood vibrancy.

Therefore, built environment indicators and socio-economic factors can significantly explain the variation of neighborhood vibrancy. Consistent built environment indicators may have different influences. Proper building density, high functional diversity, and accessibility have greater impacts with respect to creating vibrancy.

## 7. Discussions

*The Death and Life of Great American Cities* examined the elements of urban architecture, and how they function in urban life. Decades have passed, but this book still provided a framework for assessing urban vitality [4]. However, current researches lack the study of how consistent built environment factors affect urban vitality of cities with different development situations. With the advance of Information and Communication Technology, this study analyzed the correlation between urban vitality and built environment of cities with different development and construction situations. The data we used for evaluating the urban vitality and built environment were provided by different open data platforms. Thus, this article provides readers with how to use open platform data to evaluate the quality of urban space.

However, this study gave a more in-depth interpretation about Jacobs’s suggestion that good neighborhood vitality requires a dense population distribution [4], when compared with other researches [15,29,38]. They found that population density indicator contributions to the creation of neighborhood vibrancy [15,29], however, excessively dense population may suppress the creation of urban vitality in our research. Moreover, high density doesn’t mean over intensive and over-exploitation. A low *FAR* and high ground building density combination or high *FAR* and low ground building density combination do not effectively create good urban vitality. Therefore, the consistent built environment factors may contrarily contribute to vitality of cities with different development stages. It is important to conduct comparative analysis of built environment measurement system for different cities to evaluate urban vitality.

As with all studies, there were challenges in the approach. First, when applying spatial-temporal big data into human dynamic research, it is important to consider the representativeness of data [51,52], such as spatial and temporal sampling characteristics, and age, gender, occupation differences of the sampling group. In addition, the study did not identify the influence path of neighborhood vibrancy, meaning that neighborhood vibrancy may also be related to the spatial form indicators of the adjacent neighborhoods. The research did not address this point. Finally, more open data platforms are needed to measure built environment, to make the measurement system completer and more accurate.

## 8. Conclusions

This study used different open data platforms to quantitatively measure neighborhood vibrancy in the central urban areas of Beijing and Chengdu in China. This study found that neighborhood vibrancy in Beijing was significantly higher than Chengdu. Both cities exhibited clear spatial heterogeneous patterns in neighborhood vibrancy. The results of the linear regression analysis showed that socio-economic indicators accounted for approximately 30% of the neighborhood vibrancy. The excessive population density inhibits resident check-in behavior; however, there is a strong positive correlation between housing prices and neighborhood vibrancy, especially for Chengdu. Houses have better locations and infrastructure is often associated with higher prices. These places in urban environments help stimulate urban vitality.

When the built environment indicators are introduced into the regression model, the accuracy of the linear regression model increased from approximately 0.3 to 0.5. This indicates a strong correlation between built environment and neighborhood vibrancy. The entropy of function diversity and transport accessibility indicators are highly positively correlated with neighborhood vibrancy. Compact cities also advocate diverse urban land use and mix of functions to inspire the vitality of urban space. China is in a rapid urbanization stage; the difficulties associated with the mixed land use emerging from rapid urbanization still need systematic research and exploration. In addition, the density of bus stations in the central urban area of Beijing is approximately three times higher than in Chengdu. Optimizing the Chengdu transportation system from the perspective of public transport accessibility could help promote the urban vitality.

Conclusions about creating urban vitality mainly relate to the appropriate construction intensity, sufficient functional mix, and high accessibility. Moreover, this study focused on analyzing the impacts of building density and floor area ratio (*FAR*) on neighborhood vibrancy. The study found that the floor area ratio and building density play different roles, strengthening an understanding of compact city concept. In our research, we found that two different construction intensity indicators, building density indicator and floor area ratio (FAR), had different effects on the vitality of cities with different development stages. The study highlights that urban planners and managers should control the relationship between building density and *FAR*. This avoids disorderly city expansion and over-exploitation.

## Figures and Tables

**Table 1:** Characteristics of built environment variables in regulatory planning management unit (RPMU).

Measurement System	Indicator	Beijing		Chengdu		Units
		Mean	*Std*	Mean	*Std*	
Social-economic data	Population	2.41	2.62	2.26	2.47	10,000 person/km^2^
	House price	4.33	1.18	0.88	0.22	10,000 CNY/m^2^
Compactness	Area	6.69	7.71	5.97	6.96	km^2^
	RCI	0.35	0.07	0.34	0.07	
POI mixed use	Entropy	0.92	0.09	0.98	0.07	
Accessibility	BNI	0.25	0.14	0.08	0.04	100 stations/km^2^
Density	FAR	1.06	0.46	1.09	0.66	
	BDI	0.22	0.08	0.21	0.08	
	RDI	9.67	4.34	7.47	3.06	km/km^2^
Landscape	GCI	0.03	0.06	0.02	0.03	

*std* stands for standard deviation.

**Table 2:** Regression results for the impact of built environment on neighborhood vibrancy.

Indicators	Model 1		Model 2	
	Chengdu	Beijing	Chengdu	Beijing
Intercept	3.96 * (0.43)	4.74 * (0.33)	2.12 * (1.63)	3.08 * (0.92)
Population density	−0.12 * (0.04)	−0.18 * (0.03)	−0.13 * (0.05)	−0.16 * (0.03)
House Price	1.84 * (0.44)	0.47 * (0.08)	0.77 (0.54)	0.46 * (0.09)
Area			0.03 (0.02)	0.06 * (0.01)
RCI			−1.33 (2.03)	−2.09 (1.62)
Entropy			1.05 * (1.65)	1.25 * (0.99)
BNI			7.53 * (3.35)	1.63 * (0.79)
FAR			−0.38 (0.32)	0.46 * (0.25)
BDI			7.33 * (1.94)	−1.33 * (1.62)
RDI			0.03 (0.03)	0.03 * (0.02)
GCI			1.03 (2.02)	0.05 (1.42)
Adjust R^2^	0.27	0.31	0.55	0.50

Standard errors are shown in parentheses, and values with * are significant at 0.1 level.
